# Experimental study on the effect of PVP, NaCl and EG on the methane hydrates formation and dissociation kinetics

**DOI:** 10.1038/s41598-024-67485-w

**Published:** 2024-07-17

**Authors:** Kaixiang Shen, Jin Zhao, Jiawei Zhou, Zonghang Wang, Yingsheng Wang

**Affiliations:** 1 Guangzhou Marine Geological Survey, China Geological Survey, Guangzhou, 511458 China; 2 School of Mechanical Engineering, Yangtze University, Jingzhou, 434023 China; 3 National Engineering Research Center of Gas Hydrate Exploration and Development, Guangzhou, 511458 China

**Keywords:** Natural gas hydrate, Kinetic inhibitors, Hydrate inhibition, Hydrate dissociation, Production stimulation, Energy, Sustainability, Chemical physics, Carbon capture and storage, Natural gas

## Abstract

The problem of hydrate plug, low efficiency of hydrate dissociation and short production time in hydrate exploitation processes have significantly hindered the commercial viability of gas hydrate extraction. This study investigated the inhibitory effects of ethylene glycol (EG), EG + polyvinyl pyrrolidone (PVP), and EG + PVP + sodium chloride (NaCl) on methane hydrate formation through experiment. The hydrate inhibitory performance is evaluated by using differential of pressure curve, the amount of hydrate, and pressure drop values, and the effects of different temperatures, pressures, inhibitors, and injection time on hydrate dissociation are further studied. The experiment results indicate that the rank of inhibitors combination in terms of effectiveness is 5%EG + 0.5 wt%PVP + 3 wt%Nacl > 10%EG + 1 wt%PVP > 30% EG. At low-temperature conditions, 30% EG exhibits good inhibition of hydrate synthesis but poor dissociation efficiency. As temperature increases, the hydrates dissociation rate with 30% EG also increases. For the combination inhibitor system of EG, PVP, and NaCl, PVP will reduce the dissociation efficiency of hydrates, while EG and Nacl will improve the hydrate dissociation performance. For low production pressure, it is found that 10% EG + 10% NaCl have a good promotion effect on hydrate dissociation, whereas under high production pressure, 20% EG + 10% NaCl is more effective. Furthermore, injecting the inhibitors earlier enhances the dissociation of hydrates more effectively.

## Introduction

In recent years, energy shortages, and environmental problems have become the focus of world attention^1^. With the rising energy consumption, natural gas hydrate has become increasingly important in both environmental and energy supply issues. Natural Gas Hydrates (NGH) have characteristics such as high gas storage density and high combustion heat value, which is a clean and efficient energy resource and mainly distributed in permafrost regions and deep-water sedimentary layers on the continental margins^2–4^. The NGH resources in the South China Sea alone is upto 85 × 10^12^ m^3^, which is more than 2 times the total conventional natural gas reserves on land nationwide. It is a potential replacement energy source following shale gas, tight gas, and coalbed methane^5–8^. The great pressure and low temperature in the deep sea create favorable conditions for hydrate formation^9–11^. However, during NGH production by depressurization, hydrate dissociation causes JT cooling, leading to the formation of secondary hydrates and ice, blocking flow channels, and causing blockages and damage to gas production wells and transportation pipelines^12–14^. Additionally, NGH reservoirs present challenges such as low permeability, poor flowability and low production, posing significant challenges to hydrate development. Research on composite system reagents that inhibit hydrate synthesis and promote the efficient dissociation of hydrate is of great significance to prevent the blockage of hydrate, improve the seepage channel, ensure the safe flow in the formation, and increase hydrate dissociation. The inhibitors are broadly classified into two categories: thermodynamic hydrate inhibitors (THIs) and kinetic hydrate inhibitors (KHIs).

Thermodynamic hydrate inhibitors function by altering the equilibrium conditions required for hydrate formation. Common THIs include methanol and glycol, which are often used in significant quantities to ensure efficacy. These inhibitors work by disrupting the hydrogen bonding network of water molecules, thereby preventing the formation of the hydrate lattice. Historically, THIs have been widely adopted due to their reliability and effectiveness^15,16^. However, their use can be costly and environmentally detrimental due to the large volumes required.

Kinetic hydrate inhibitors, on the other hand, do not change the thermodynamic conditions of hydrate formation. Instead, KHIs delay the formation of hydrate crystals and slow down their growth rate, providing a temporal window during which the gas can be transported without the risk of blockage. These inhibitors are typically polymers or surfactants that adsorb onto the surface of hydrate nuclei, hindering their growth. KHIs are used in much lower concentrations than THIs, making them a more cost-effective and environmentally friendly option. However, their effectiveness can be influenced by temperature, pressure, and the composition of the gas and water phases^17–21^.

Currently, study on inhibiting hydrate synthesis mainly involves experimental methods and molecular dynamics simulation methods^22–27^. Carver utilized molecular simulations to study the effect of PVP on the hydrate surface, and found that PVP was mainly bonded to hydrate by hydrogen bond of amino group and molecular force between ring and hydrate surface, thereby inhibiting hydrate growth^28^. Walsh simulated the nucleation and growth processes of natural gas hydrates at the microsecond scale^29^. Reilly was first to study the performance of poly (N-vinyl piperidone) (PVPip) to inhibit the growth of hydrate crystal^30^. Kamal reviewed the performance of various hydrate inhibitors and the techniques used to evaluate the performance of different inhibitors^31^. Nanoparticles such as zinc oxide and graphite, have also been widely used to prevent the agglomeration of the hydrate particles due to its high surface area and unique physicochemical properties^32^. Liu et al. reported the effect of SDS-coated Fe_3_O_4_ nanoparticles on the hydrate formation, which had less induction time than standalone SDS^33^. Alef and Barifcani investigated the effect of N-methyl-diethanolamine and film forming corrosion inhibitor on gas hydrate and developed two empirical models to cater for MEG and MDEA + MEG degradation effect on hydrate phase equilibrium temperature^34^. Nasir presented a comprehensive review on the different chemical additives used to promote or inhibit the hydrate formation and analyzed the challenges faced by the inhibitor development^35^. Liao et al. used the experiment and molecular simulation method to reveal the mechanisms of alkylated hydrate inhibitor, SYZ-2 and its NaCl system on hydrate nucleation and formation^36–38^. The hydrate inhibition is achieved by reducing the solubility of methane molecules in water during the nucleation phase and by adsorption during the formation phase^39–43^. Dai investigated the effects of alcohol chain length, hydroxyl group position, and hydroxyl group number on methane hydrate dissociation based on molecular dynamics simulations, and found that shorter chain length alcohols promote methane hydrate dissociation, while shortening the alcohol chain length and increasing the number of hydroxyl groups facilitate methane hydrate dissociation^44^. During hydrate synthesis and dissociation experiment, real-time detection and quantitative analysis are challenging^45–50^. Additionally, due to limitations in research methods, scholars have yet to reach a consensus on the nucleation mechanism of hydrates^51–54^. Currently, the micro-mechanisms of kinetic inhibitors and hydrate dissociation remain unclear.

This study aims to assess the effectiveness of different inhibitors and explore the influence of experimental conditions on hydrate formation and dissociation. Initially, the effect of various concentrations of EG, EG + PVP, and EG + PVP + NaCl on hydrate synthesis were investigated. Subsequently, the impact of temperature, pressure, and injection time on hydrate dissociation was also investigated. The mechanism of hydrate synthesis and dissociation was revealed by experiments, and the cost-effective inhibitor combination and hydrate dissociation promoter were also identified. These findings underscore the importance of inhibitor concentration, temperature, and injection timing in optimizing hydrate development. Achieving the ideal balance of chemical additive concentrations and experimental conditions is crucial for effective hydrate inhibition and gas production enhancement. Future research endeavors could provide further insights into enhancing gas production efficiency and offer practical implications for industrial applications.

## Methodology

### Materials

Poly (vinyl pyrrolidone) (PVP) (CAS:9003-39-18, average Mw 8000), ethylene glycol (CAS:107-21-1, 98% purity), sodium chloride (CAS:7647-14-5, 98% purity) were obtained from MACKIN Reagent Co., Ltd. (Shanghai, China). Methane gas (99% purity) was obtained from Wuhan Xinxing Industrial Gas Co., Ltd. (Wuhan, China). Deionized water, as a solvent, was made in the laboratory with a resistivity of 18.25 mΩ cm at 25 ℃.

### Apparatus

**Figure 1:**
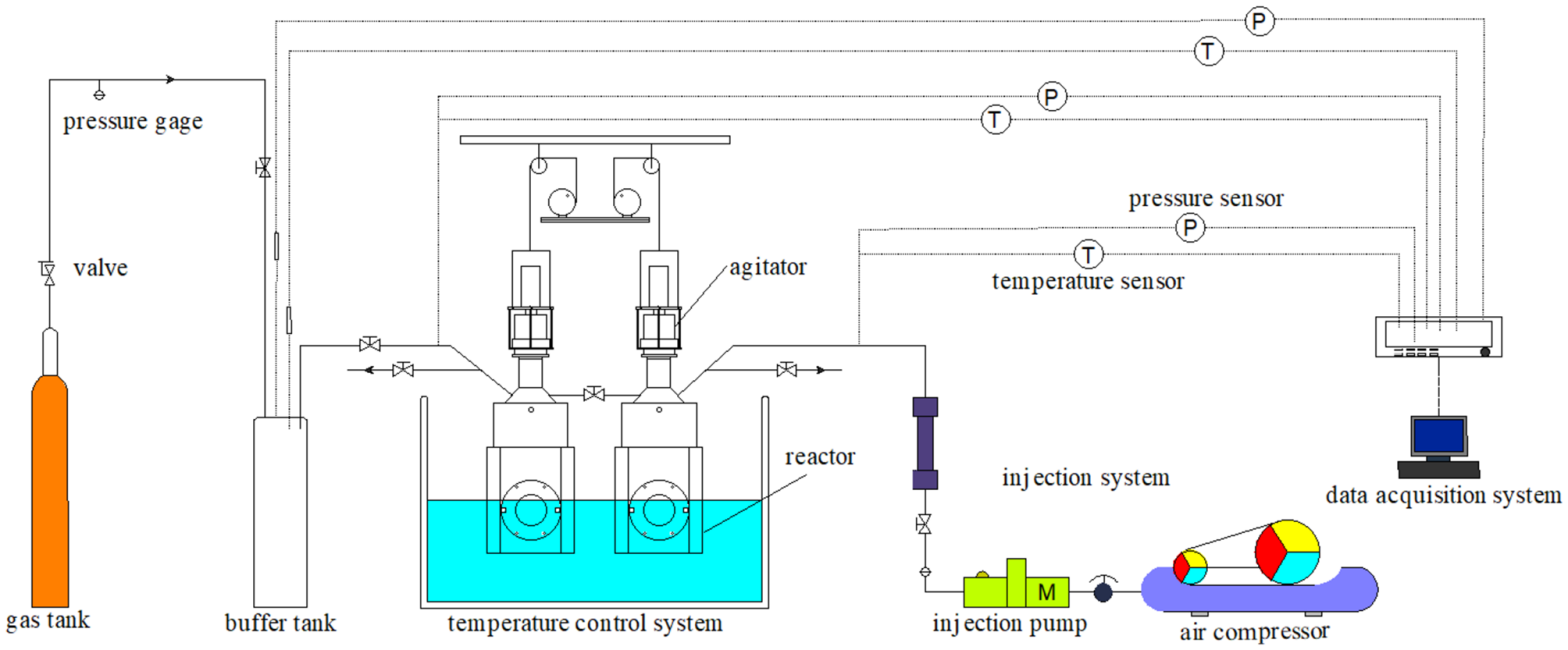
Hydrate formation and dissociation experimental process diagram.

The visualization and simulation apparatus for hydrate formation and dissociation mainly consists of gas tank, gas pressurization system, constant temperature cooling water bath system, control panel with data acquisition system, and system for hydrate synthesis and inhibitor injection, as depicted in Fig. 1.

The volume of the reaction vessel is 500 ml, with a maximum working pressure of 25 MPa and a working temperature range of − 10 °C to 100 °C. Inside the reaction vessel, there is an agitator with a maximum speed of 500 rpm, which enhances the speed of hydrate synthesis. Visual windows are located at both ends of the reaction vessel to facilitate real-time observation of hydrate formation and dissociation processes. Various chemical additives can be injected into the reactor through the inhibitor injection system, allowing for the simulation of diverse inhibitor effects on the dissociation of hydrates. The temperature control system enables gradient heating and cooling of the water bath, while the data acquisition system allows real-time monitoring of various parameters during the experiment, facilitating analysis of hydrate formation and dissociation processes.

### Experimental procedure

To address the issues of long natural gas hydrate synthesis time and low hydrate saturation under static conditions, the formation of hydrates is accelerated by stirring. Prior to the dissociation experiment, hydrate is synthesized, and the inhibitor is introduced into the reaction vessel through an intermediate container to ensure sufficient contact with the hydrate. The specific experimental procedure is as follows:

- (1) Prepare the inhibitor concentration as required and add it to the reactor, then assemble the experimental setup.
- (2) Utilize the water bath circulation system to cool the reactor until the temperature reaches the 3 °C and maintain the temperature.
- (3) Open the gas cylinder and introduce methane gas into the reactor to check the sealing integrity.
- (4) Once the gas-tightness test is finished, open the gas cylinder valve and inject methane gas into the reactor to achieve a pressure of 15 MPa, simultaneously initiating data collection to record experimental parameters such as temperature and pressure.
- (5) When the temperature and pressure is stable, activate the magnetic stirrer at a speed of 300 rpm. Temperature, pressure, and stirring rate data will be displayed in real-time on the computer. The hydrate synthesis experiment should last for at least 10 h, and completion is indicated when the pressure stabilizes.
- (6) After hydrate synthesis, depressurize the reactor to 5 MPa, then use the injection system to evenly introduce chemical additives into the reactor, set the right production pressure and temperature, and commence the hydrate dissociation simulation experiment.
- (7) After the experiment, the pressure relief valve of the reaction vessel is slowly opened to release the pressure, and the remaining solution is poured out. The reaction vessel is cleaned, and temperature, pressure, and other data are saved.

### Calculation of gas consumption and hydrate formation

The reaction is conducted in an isochoric reaction vessel, and the gas consumption can to some extent reflect the formation of hydrates. The gas consumption during a certain period can be calculated using the actual gas state Eq. ^55^,

1 $${m}_{g}=(\frac{{P}_{0}{V}_{0}}{{Z}_{0}R{T}_{0}}-\frac{{P}_{t}{V}_{t}}{{Z}_{t}R{T}_{t}})\times 16$$

where *m*_*g*_ is the gas consumption, Kg; *P* is the gas pressure, Pa; *V* is the gas volume, m^3^; *T* is the gas temperature, *T* = 273.15 + t (T is in Kelvin and t is in Celsius); *R* is the molar gas constant, J/(mol·K), R = 8.314; *Z* is the compressibility factor, which is related to both temperature and pressure, with subscripts 0 and t representing the initial time and final time respectively.

The total methane consumption during the hydrate experimental reaction process can be assumed to participate in the hydrate reaction. The amount of hydrate formation during the reaction process is given by:

2 $${m}_{s}=\frac{\frac{{P}_{0}{V}_{0}}{{Z}_{0}R{T}_{0}}-\frac{{P}_{t}{V}_{t}}{{Z}_{t}R{T}_{t}}}{\frac{{P}_{0}{V}_{0}}{{Z}_{0}R{T}_{0}}-\frac{{P}_{e}{V}_{e}}{{Z}_{e}R{T}_{e}}}{m}_{0}$$

where m_0_ is the total mass of hydrates generated after the experiment ends, kg; m_s_ is the mass of hydrates generated by the system, kg; the subscript e denotes the experiment end time.

## Results and discussion

To prevent hydrate formation during production and enhance nature gas production, it is essential to investigate both the mechanism of inhibiting hydrate synthesis and promoting dissociation. Initially, we examine the impact of various inhibitors on hydrate synthesis. Subsequently, employing a visualization simulation apparatus, we explore the effects of diverse temperatures, production pressure, inhibitors, and injection time on augmenting hydrate dissociation.

### Effects of different inhibitors on hydrate synthesis

Different concentrations of EG are examined for their impact on hydrate synthesis. Subsequently, the influence of various combinations of EG + PVP on hydrate is explored based on experimental findings. Further investigation is conducted into the effects of EG + PVP + NaCl on hydrate synthesis. Ultimately, an economical hydrate inhibitor is identified.

#### Effects of EG concentration on hydrate synthesis

**Figure 2:**
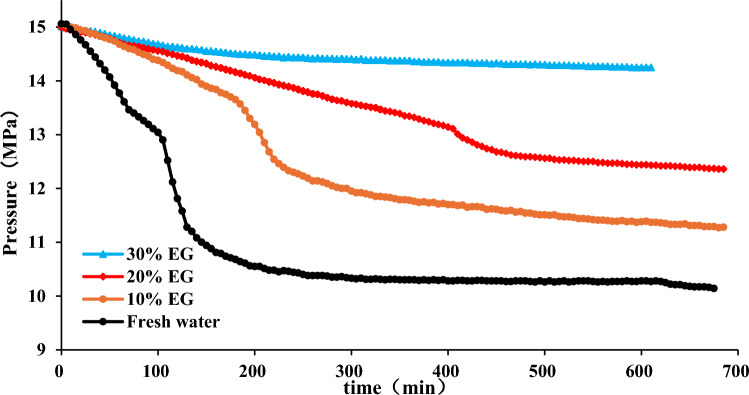
Effect of EG concentration on hydrate synthesis.

**Figure 3:**
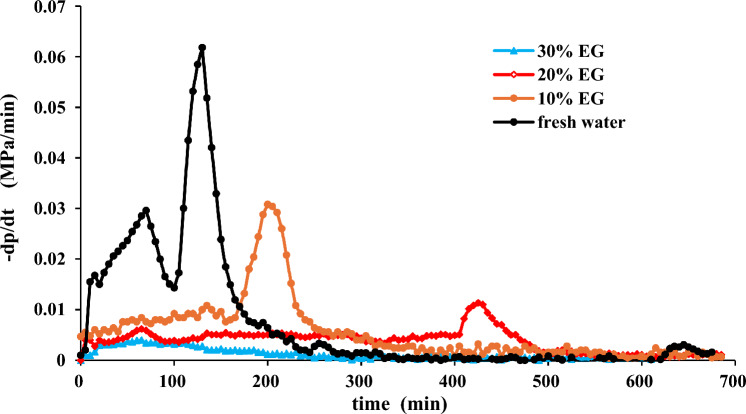
Effect of EG concentration on pressure decline rate.

**Figure 4:**
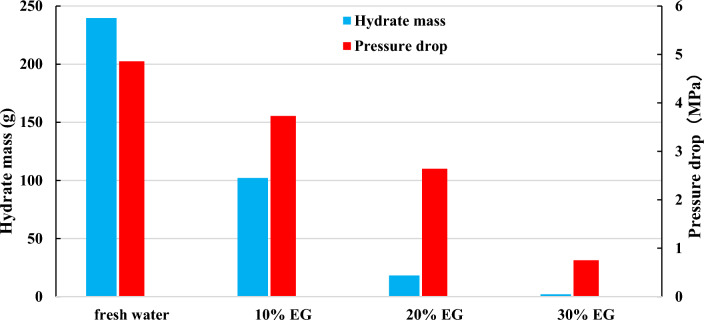
Effect of EG concentration on hydrate quality.

The experiment is conducted with an initial pressure of 15 MPa, maintaining a constant temperature of 3 °C in the water bath. The liquid volume stands at 250 mL, with the stirrer speed set to 300 rpm, and the experiment duration spans 650 min. Figure 2 depicts the impact of varying EG concentrations on hydrate synthesis. Correspondingly, the final quantity of hydrate synthesis, the resultant images, and hydrate formation results of different EG concentrations the are portrayed in Figs. 3 and 4, respectively.

Figures 2 and 3 reveal that in the case of pure water, the pressure in the reaction vessel gradually diminishes throughout the hydrate synthesis process. Between the durations of 100 to 135 min, there is an escalation in the rate of pressure reduction, culminating in a pinnacle of pressure derivative, subsequently followed by a stabilization of pressure. This phenomenon signifies an augmentation in methane gas consumption, resulting in an accelerated pace of hydrate formation and a consequent decline in pressure. Conversely, with an elevation in EG concentration, the peak of the pressure derivative undergoes a delay, leading to a reduction in the rate of pressure decay and a progressive inhibition of hydrate synthesis.

**Figure 5:**
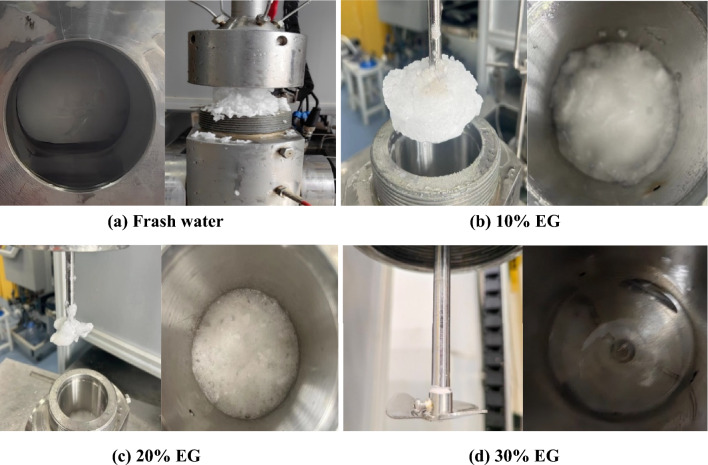
Hydrate formation results of different EG concentration (**a**) Frash water (**b**) 10% EG (**c**) 20% EG (**d**) 30% EG.

Figure 4 illustrates that with the escalation of EG concentration, both the volume of hydrate synthesis and the reduction in pressure within the reaction vessel diminish. This phenomenon arises from the heightened concentration of EG, which impedes hydrate synthesis, consequently leading to a decreased consumption of methane gas within the reaction vessel and a consequent attenuation in pressure reduction. At a concentration of 30%, the pinnacle of the pressure derivative vanishes, resulting in a negligible pressure drop and little formation of hydrate, thereby indicating that 30% EG has a better inhibitory effect on hydrate synthesis. Figure 5 demonstrates that the higher the ethylene glycol concentration is, the less hydrate synthesis will be. It is worth mentioning that EG is toxic and can pose environmental hazards if spilled or improperly disposed of. Its use requires careful handling and disposal to mitigate environmental risks^56,57^.

#### Effects of combinations of EG and PVP on hydrate synthesis

**Figure 6:**
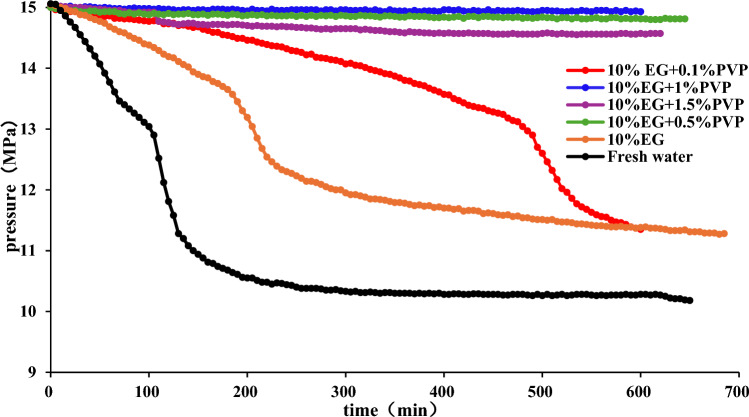
Effect of 10%EG + PVP on hydrate formation.

**Figure 7:**
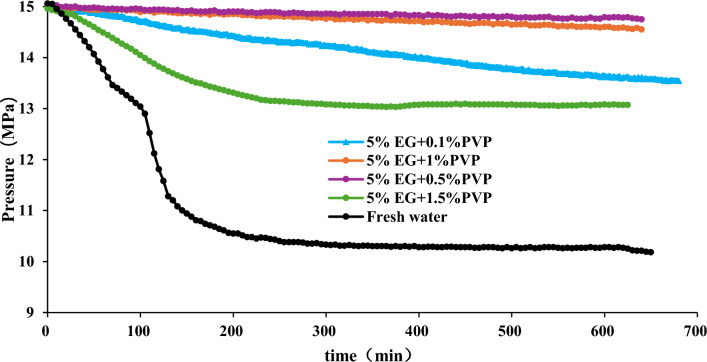
Effect of 5%EG + PVP on hydrate formation.

**Figure 8:**
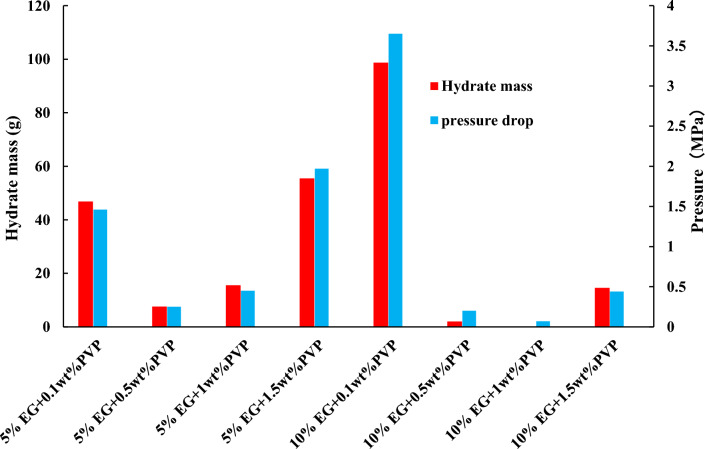
Hydrate formation weight and pressure drop of different inhibitors.

To reduce the concentration of EG and obtain an economic inhibitor, the effects of binary combinations of 5%, 10% EG and 0.1%, 0.5%, 1%, 1.5% PVP on hydrate synthesis are studied. The experiment results are presented in the Figs. 6–8.

Figure 6 indicates that compared to pure EG, the addition of PVP prolongs the rapid pressure drop stage, suggesting that the addition of PVP can effectively inhibit hydrate synthesis. When the EG concentration is 10%, elevating the PVP concentration from 0.1 to 1% diminishes methane consumption, leading to an increase in the ultimate pressure within the reaction vessel. However, with further increasing PVP concentrations, pressure declines while methane utilization rises, leading to a less favorable inhibition of hydrate formation.

Figures 7 and 8 illustrate that with a 5% EG concentration, raising the PVP concentration from 0.1 to 0.5% results in an increase in final pressure in the reaction vessel, a reduction in hydrate occurrence, and an enhancement in the inhibitory efficiency. However, further escalation of PVP concentration leads to a decline in stable pressure in the reaction vessel, an increase in pressure drop, heightened hydrate formation, and diminished inhibitory effect. In the binary inhibition, 10% EG + 1wt% PVP exhibits the most potent inhibitory prowess with no hydrate formation, whereas 10% EG + 0.5wt% PVP and 5% EG + 0.5wt% PVP yield a minor amount of hydrate formation.

#### Effects of combinations of EG, PVP, and NaCl on hydrate synthesis

**Figure 9:**
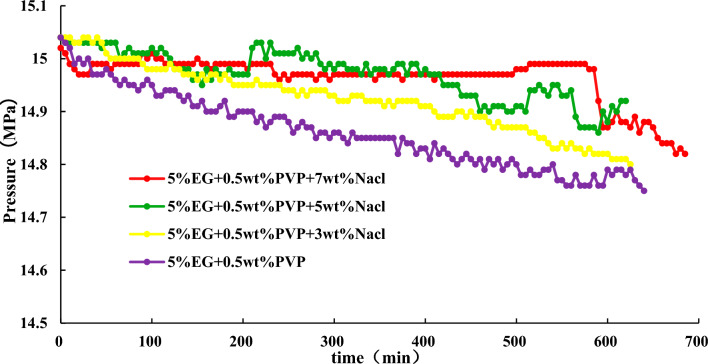
Effect of combination of NaCl, PVP and EG inhibitors on hydrate synthesis.

To further enhance the inhibitory effect of inhibitors, the effects of 5% EG + 0.5wt% PVP + 3%, 5%, 7% NaCl on hydrate synthesis are studied based on the above experimental results. The results are shown in the Fig. 9.

Figure 9 illustrates that the inclusion of NaCl leads to a gradual rise in stable pressure within the reaction vessel, a reduction in hydrate consumption, and an augmentation of the inhibitory influence on hydrate synthesis. Notably, no hydrates form within the reaction vessel, and the pressure decline within it remains below 0.2 MPa. Furthermore, as the concentration of NaCl increases, so does the inhibitory efficacy of the inhibitor. Therefore, opting for 5% EG + 0.5 wt% PVP + 3 wt% NaCl can effectively impede the synthesis of hydrates, which can be used as an economic inhibitor.

### Factors Influencing hydrate dissociation

Hydrate dissociation has great influence on gas production. To improve gas production, understanding the influence of parameters such as temperature, pressure, inhibitors, and injection time on dissociation allows us to develop strategies for effective hydrate management in various industrial applications, including energy production, gas transportation, and climate change mitigation. Therefore, to make the mechanism of hydrate dissociation clear, NGH is first performed by an experimental device, and then the effect of different temperature, production pressure, various inhibitors, and injection time on hydrate dissociation is investigated.

#### Effect of temperatures on hydrate dissociation

**Figure 10:**
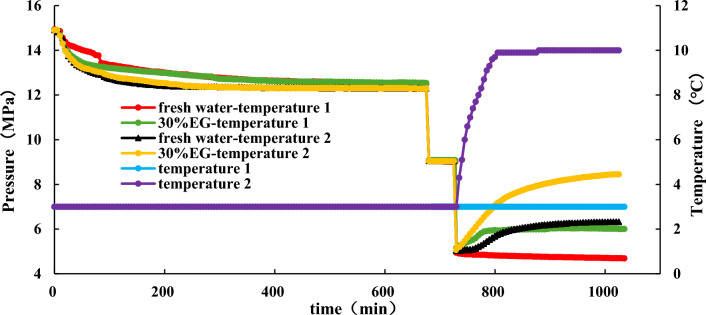
Effect of temperature on hydrate dissociation.

NGH is first synthesized under conditions of 15 MPa and 3 °C. Once the pressure in the reaction vessel is stable, reduce it to 9 MPa and maintain it for one hour. Then, the hydrate dissociation experiment is carried out. During the experiment, Reaction Vessel 1 is kept at a constant temperature of 3 °C, while Reaction Vessel 2 gradually increases from 3 °C to 10 °C and then stabilized. The pressure and water bath temperature inside the reaction vessel during dissociation are shown in Fig. 10.

**Figure 11:**
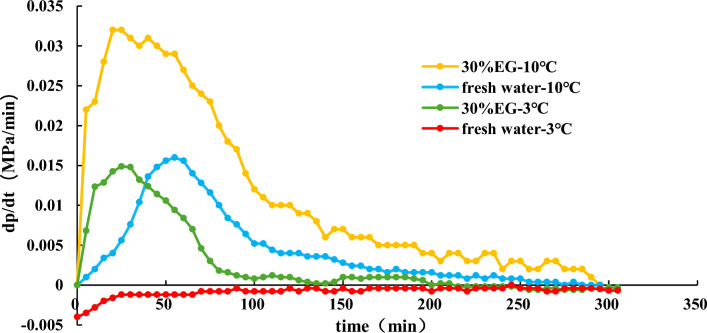
Effect of temperature on gas pressure derivative during hydrate dissociation.

Figure 10 and 11 indicate that it takes 80 min for the water bath temperature to ascend from 3 °C to 10 °C. With this rise in temperature, there is an observed escalation in the pressure derivative, signifying accelerated hydrate dissociation. In the case of pure water at 3 °C, the pressure derivative remains negative, indicating a slow formation of hydrates. Conversely, the introduction of EG causes the pressure derivative to rise, implying accelerated hydrate dissociation and an elevation in pressure within the reaction vessel. This indicates that under 3 °C conditions, 30% EG can enhance hydrate dissociation, albeit temporarily and with a limited pressure increase, resulting in subdued gas production.

A comparison of the experimental data for EG at 10 °C and 3 °C reveals that augmenting the temperature leads to a greater pressure increase than adding ethylene glycol, suggesting that elevating the temperature yields superior production enhancement compared to employing ethylene glycol. Furthermore, for identical EG concentrations, increasing the temperature amplifies its impact on hydrate dissociation.

#### Effect of production pressure on hydrate dissociation

**Figure 12:**
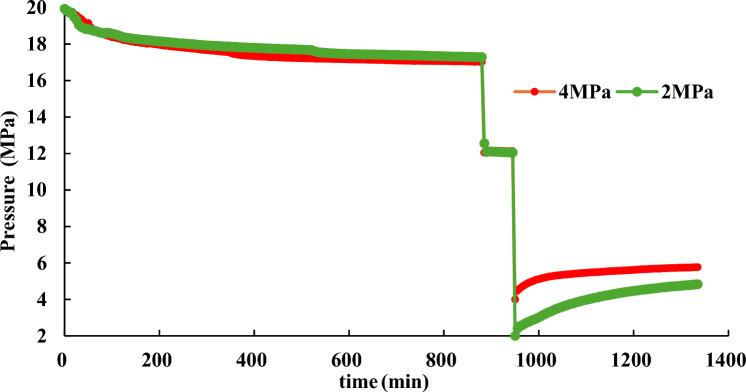
Effect of production pressure on hydrate dissociation.

**Figure 13:**
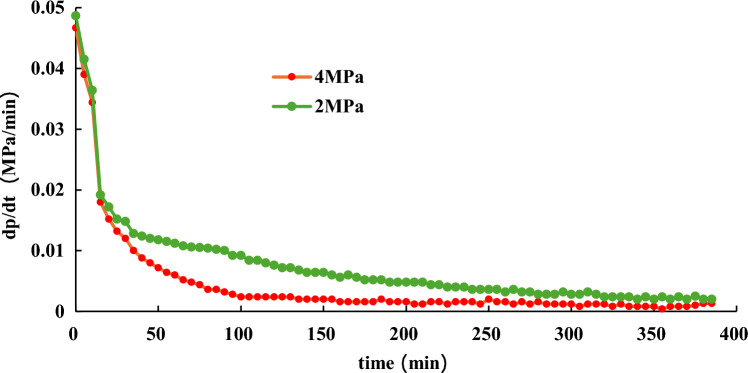
Effect of production pressure on gas pressure derivative during hydrate dissociation.

To simulate the actual process of hydrate dissociation, NGH is formed under conditions of 20 MPa and 10 °C. Once the pressure in the reaction vessel is stable, maintain it for one hour before reducing it to 12 MPa. To investigate the effect of production pressure on the rate of hydrate dissociation, the pressure of the 1# reactor is reduced to 4 MPa, and 2# reactor is reduced to 2 MPa. The water bath temperature is set to 10℃ during the whole process. Experiment results are presented in Fig. 12, 13.

Figure 12 depicts that during hydrate formation process, the final pressure are 17.3 MPa and 17 MPa, respectively, which means that there is little difference in hydrate synthesis weight between the two reactors. To keep the consistent experimental conditions before hydrate dissociation experiment, the pressure in the reaction vessel is first reduced to 12 MPa, and maintain it for one hour. The effects of production pressure on hydrate dissociation are shown in Fig. 13.

Figures 12 and 13 demonstrate that during the hydrate dissociation process, as the production pressure decreases, there is a corresponding increase in the pressure derivative, indicating a faster rate of hydrate dissociation and a more rapid rise in pressure inside the reaction vessel. As time progresses, the pressure derivative gradually diminishes, indicating a deceleration in the rate of hydrate dissociation. Hence, reducing reservoir pressure can promote the dissociation of hydrates.

#### Effect of different inhibitors on hydrate dissociation

**Figure 14:**
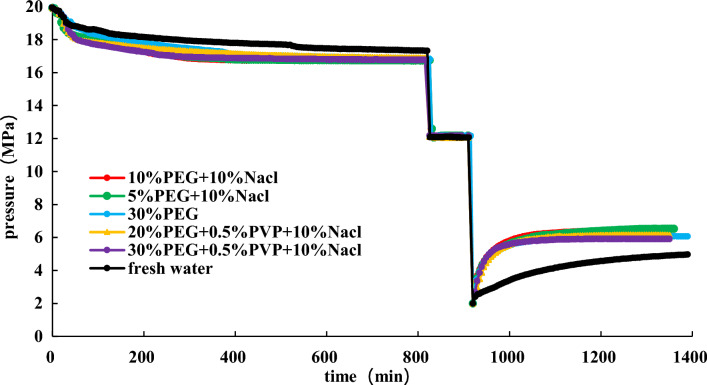
Effect of different inhibitors on hydrate dissociation with initial pressure 2 MPa.

**Figure 15:**
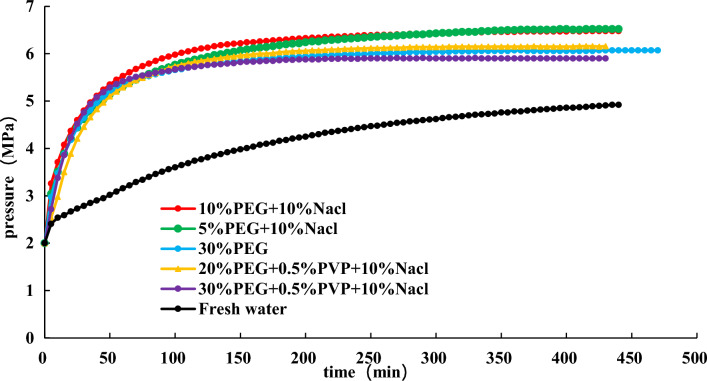
Effect of different inhibitors on gas pressure during hydrate dissociation at initial pressure of 2 MPa.

**Figure 16:**
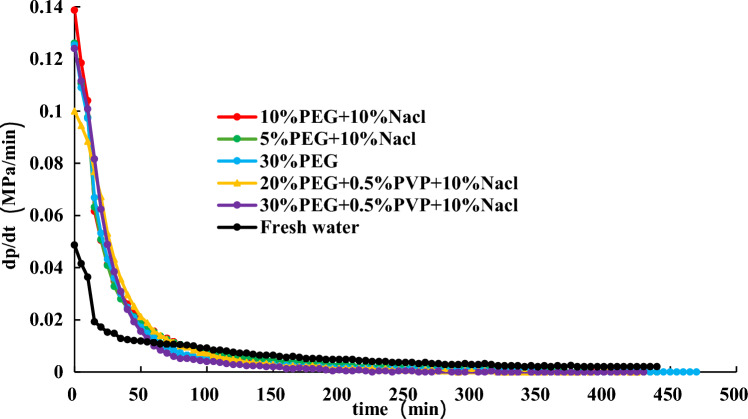
Effect of different inhibitors on gas pressure derivative during hydrate dissociation at initial pressure of 2 MPa.

To find a more economical accelerator of hydrate dissociation, following the above experimental steps in Section “Effect of production pressure on hydrate dissociation”, the effect of 30% EG + 0.5 wt% PVP + 10 wt% NaCl, 20% EG + 0.5 wt% PVP + 10 wt% NaCl, and 30% EG on hydrate dissociation are studied at an initial pressure of 2 MPa. The experimental findings are shown in Figs. 14, 15, 16.

Figures 14, 15, 16 present that utilizing the method of significant depressurization for hydrate exploitation (initial production pressure of 2 MPa), 30% EG + 0.5 wt% PVP + 10 wt% NaCl exhibits lower final pressure and pressure derivative compared to 30% EG alone, resulting in a diminished effect on gas production. According to the final pressure and the change in pressure derivative in the reaction vessel, it is evident that 10% EG + 10 wt% NaCl demonstrates the most favorable production enhancement effect, followed by 10% EG and 5 wt% NaCl. This indicates that the introduction of PVP within the ternary system decelerates the rate of hydrate dissociation, thereby impeding gas production. Consequently, utilizing the EG and NaCl combination inhibitors prove more effective for hydrate dissociation. Injecting chemical additive within 50 min results in a noticeable effect on hydrate dissociation, characterized by a significant and rapid decrease in pressure derivative, leading to accelerated pressure rise within the reaction vessel. Subsequently, pressure increase decelerates after 50 min, with the pressure derivative reaching stabilization. Ultimately, after 360 min, pressure inside the reaction vessel gradually stabilizes.

**Figure 17:**
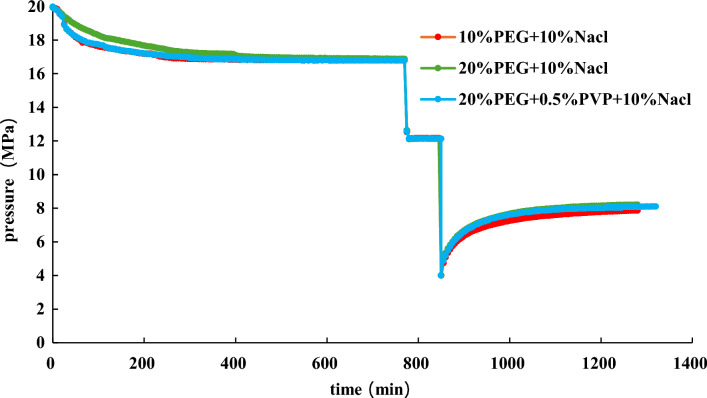
Effect of different inhibitors on hydrate dissociation with initial pressure 4 MPa.

**Figure 18:**
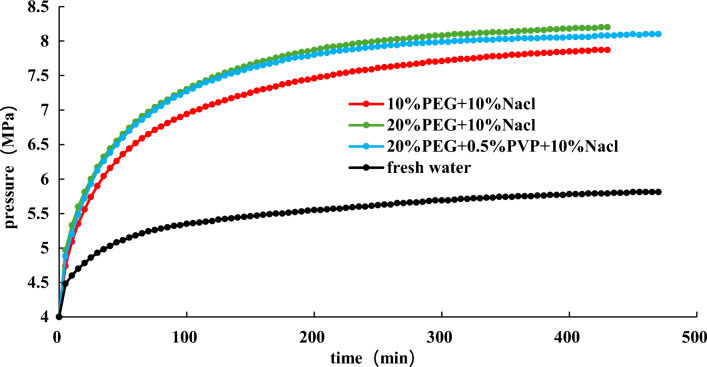
Effect of different inhibitors on gas pressure with initial pressure 4 MPa during the hydrate dissociation process.

**Figure 19:**
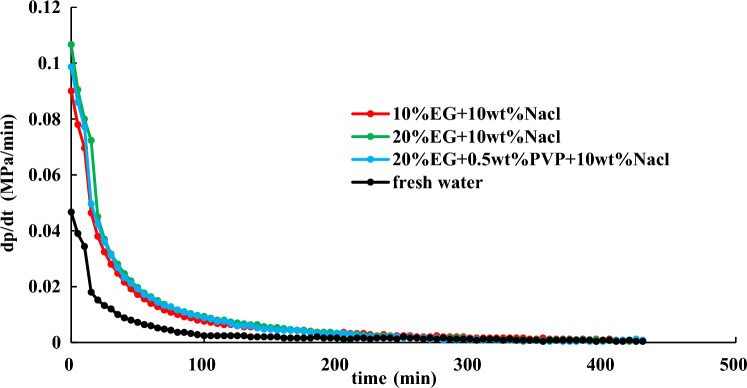
Effect of different inhibitors on gas pressure derivative during hydrate dissociation at initial pressure of 4 MPa.

Base on the above experiments, the effects of 20% EG + 10 wt% NaCl, 20% EG + 0.5% PVP + 10 wt% NaCl, and 10% EG + 10 wt% NaCl are further studied at an initial pressure of 4 MPa. The experimental results are demonstrated in the Fig. 17, 18, 19.

Figures 17, 18, 19 illustrate that during slightly depressurization process (initial production pressure of 4 MPa), the most effective production enhancement is observed with 20% EG + 10wt% NaCl, followed by 20% EG + 0.5 wt% PVP + 10 wt% NaCl, while 10% EG + 10 wt% NaCl shows the least improvement in production. This reaffirms the notion that the addition of PVP diminishes the hydrate dissociation. For slight depressurization method, increasing the concentration of EG proves beneficial, particularly with 20% EG + 10 wt% NaCl offering optimal production enhancement.

#### Effect of inhibitor injection time on hydrate dissociation

**Figure 20:**
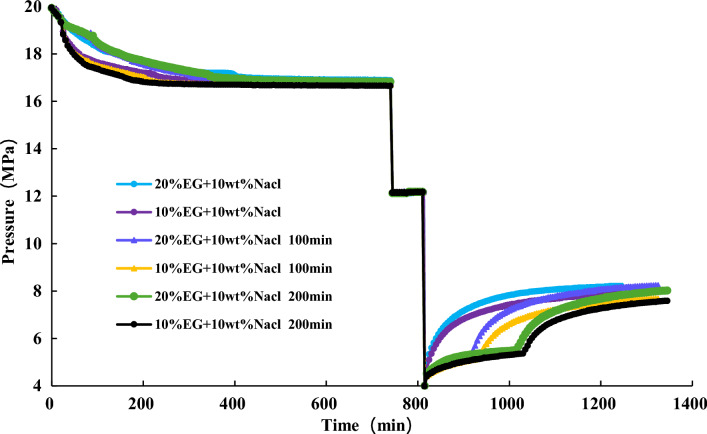
Effect of inhibitor injection time on hydrate dissociation.

**Figure 21:**
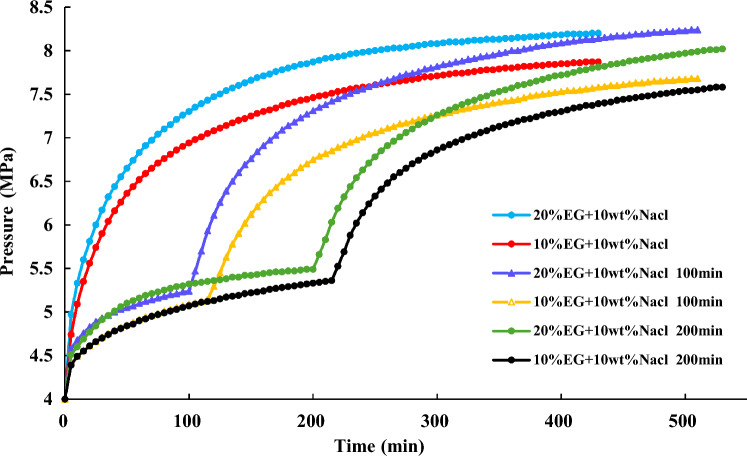
Pressure variation during the hydrate dissociation process.

NGH is first synthesized under conditions of 20 MPa and 10 °C. Once the pressure in the reaction vessel is stable, maintain it for one hour before reducing it to 12 MPa. Subsequently, decrease it to 4 MPa. After hydrate dissociation at 0 min, 100 min, and 200 min, inject 20% EG + 10% NaCl and 10% EG + 10% NaCl, respectively, and compare their effects on hydrate dissociation. The experimental results findings are illustrated in Fig. 20 and 21.

Figures 20 and 21 indicate that after the injection of inhibitors, there is a rapid dissociation of hydrates, leading to a rapid increase in pressure. The earlier the inhibitors are injected, the quicker the effect is observed. At the beginning of hydrate depressurization, injecting 20% EG + 10% NaCl will have a good effect on gas production. For the combination of EG and NaCl inhibitors, when the NaCl concentration is 10%, a higher concentration of EG yields better gas production enhancement.

## Conclusion

This study experimentally investigates the inhibitory effects of EG, EG + PVP, and EG + PVP + NaCl on methane hydrate formation. Additionally, the influence of various temperatures, production pressure, inhibitors combination and injection time on methane hydrate dissociation is examined. The findings underscore the intricate interplay of inhibitor concentrations, temperature, production pressure and injection timing in optimizing gas production from methane hydrate. These insights provide guidance for designing efficient hydrate inhibition strategies in the oil and gas industry. The main conclusions are as follows:

- (1) Increasing ethylene glycol concentrations will inhibit methane hydrate formation. A concentration of 30% ethylene glycol exhibits good synthesis inhibition. However, higher concentrations of both EG and PVP in combination do not necessarily lead to better synthesis inhibition. Effective inhibition of methane hydrate formation can be achieved by using 10% EG + 1% wt PVP or 5% EG + 0.5 wt% PVP + 3 wt% NaCl.
- (2) In low-temperature settings, 30% EG demonstrates a better inhibitory effect on methane hydrate synthesis lower efficiency in dissociation. Nevertheless, increasing the temperature can enhance the efficiency of EG in decomposing hydrates. Raising the temperature or reducing production pressure can enhence the pressure derivative, thereby improving hydrate dissociation.
- (3) The most significant enhancement in production occurs within the first 100 min after injecting inhibitors, gradually weakening thereafter. When combined with 10% NaCl, higher concentration of EG leads to better gas production from methane hydrate.
- (4) For inhibitors combining EG, PVP, and NaCl, the addition of PVP diminishes the gas production. Conversely, increasing the concentrations of EG and NaCl can amplify the gas production. When the production pressure is low, it is recommended to use 10% EG + 10% NaCl for better dissociation outcomes. Conversely, under higher production pressure conditions, utilizing 20% EG + 10% NaCl yields superior dissociation. Moreover, the earlier the inhibitors are injected, the more effective the dissociation of methane hydrates becomes.

## Data Availability

The data presented in this study are available from the corresponding author on reasonable request.
